# Targeted LC-MS Orbitrap Method for the Analysis of Azaarenes, and Nitrated and Oxygenated PAHs in Road Paving Emissions

**DOI:** 10.3390/molecules30163397

**Published:** 2025-08-16

**Authors:** Maria Bou Saad, Sylvain Ravier, Amandine Durand, Brice Temime-Roussel, Vincent Gaudefroy, Audrey Pevere, Henri Wortham, Pierre Doumenq

**Affiliations:** 1 LCE, Aix Marseille University, 13331 Marseille, France; sylvain.ravier@univ-amu.fr (S.R.); amandine.durand@univ-amu.fr (A.D.); brice.temime-roussel@univ-amu.fr (B.T.-R.); pierre.doumenq@univ-amu.fr (P.D.); 2 Materials for Transportation Infrastructures Laboratory (MIT), Materials and Structures Department (MAST), Université Gustave Eiffel, Allée des Ponts et Chaussées, CS4, 44344 Bouguenais, France; vincent.gaudefroy@univ-eiffel.fr; 3 Cerema, University Gustave Eiffel, UMR MCD, 13100 Aix-en-Provence, France; audrey.pevere@cerema.fr

**Keywords:** PAH derivatives, LC-HRMS, trace-level quantification, azaarenes, nitrated PAH, oxygenated PAH

## Abstract

Polycyclic aromatic hydrocarbon (PAH) derivatives, specifically azaarenes and nitrated and oxygenated PAHs, are emerging contaminants of concern due to their increased toxicity and persistence compared to the parent PAHs. Despite their toxicity, their simultaneous analysis in complex matrices, such as in fumes emitted from bituminous mixtures, remains challenging due to limitations of conventional analytical techniques. To address this, an advanced methodology was developed using Ultra-High-Performance Liquid Chromatography coupled with High-Resolution Mass Spectrometry (UHPLC-HRMS Orbitrap Eclipse) equipped with an APCI source for the simultaneous identification and quantification of 14 PAH derivatives. Chromatographic and ionization parameters were optimized to ensure maximum sensitivity and selectivity. Following ICH Q2(R2) guidelines, the method was validated, demonstrating excellent linearity (R^2^ > 0.99), high mass accuracy (≤5 ppm), strong precision (<15%), and excellent sensitivity. Limits of detection (LODs) ranged from 0.1 µg L^−1^ to 0.6 µg L^−1^ and limits of quantification (LOQs) ranged from 0.26 µg L^−1^ to 1.87 µg L^−1^. The validated method was successfully applied to emissions from asphalt pavement materials collected on quartz filters under controlled conditions, enabling the identification and quantification of all 14 targeted compounds. These results confirm the method’s robustness and suitability for trace-level analysis of PAH derivatives in complex environmental matrices.

## 1. Introduction

Polycyclic aromatic hydrocarbons (PAHs) and their derivatives, such as azaarenes (aza-PAHs) or nitrogen-containing PAHs, nitrated PAHs (nitro-PAHs), and oxygenated PAHs (oxy-PAHs), are persistent organic pollutants with significant environmental and health impacts. While most studies have focused solely on the 16 parent PAHs classified by the US Environmental Protection Agency (EPA) due to their carcinogenicity and environmental significance [1,2], there is growing knowledge that their derivatives might exhibit even greater toxicity [3]. Several studies have shown that aza-PAHs, oxy-PAHs, and nitro-PAHs present higher mutagenic and reprotoxic effects than their parent compounds, as demonstrated by different bioassays, including the forward mutation assay based on human B-lymphoblastoid cells [4], the Mutatox test [5], and the Ames Salmonella test [6]. In addition to causing oxidative stress by generating reactive oxygen species [7], oxy-PAHs exhibit greater environmental persistence than other transient organic compounds, making them increasingly significant in environmental studies [8,9,10].

PAHs are primarily emitted through the incomplete combustion of fossil fuels and other organic matter [11,12,13]. In addition, PAHs can also be released from petrogenic sources, such as the fumes emitted during the production, application, and aging of asphalt materials, due to the high temperature involved in these processes [14,15]. According to the International Agency for Research on Cancer (IARC) and the World Health Organization (WHO) [16], occupational exposure to straight-run bitumen and its emissions during road paving has been classified as “possibly carcinogenic to humans” in 2011 (Group 2B). The analysis of fumes emitted from bituminous mixtures presents certain challenges due to their complex composition, which includes a wide variety of organic compounds such as volatile and semi-volatile organic compounds (VOCs and SVOCs) and PAHs and their derivatives, such as aza-PAHs, nitro-PAHs, and oxy-PAHs [17,18,19]. These compounds require advanced analytical methods for effective identification and quantification.

Traditional methods, such as HPLC-UV and fluorescence detection, are usually used for PAH analysis [20,21,22]. This approach can seem effective when analyzing the 16 parent PAHs or those of low molecular weight. However, considering the nature of nitro- and oxy-PAHs, being of higher polarity and thermolability, this method can be limited. Additionally, nitro-PAHs are challenging to analyze using fluorescence-based detection due to their lack of inherent fluorescence [22,23].

Another conventional approach is that using GC-MS and GC-MS/MS, which, due to their high resolution, can be applied efficiently [10,20,24]. Nevertheless, a loss of sensitivity is observed when analyzing compounds with low volatility and/or high polarity [25]. In addition, a thorough cleanup step is crucial when analyzing complex matrices to avoid disruptions [26]. However, this process can be time-consuming and may result in analyte losses, particularly through solvent evaporation.

More recently, liquid chromatography coupled with mass spectrometry (LC-MS) has demonstrated improved sensitivity and selectivity when analyzing PAH derivatives. Nevertheless, in many cases, researchers have used this method with two different columns, depending on the class of PAH derivatives, limiting their simultaneous analysis [15]. Additionally, while Nyiri et al. [27] addressed the complexity of PAH mixtures by using GC-MS for oxy-PAHs and LC-MS/MS for nitro-PAHs, concurrent analysis of these compounds using high-resolution LC-MS remains scarce in complex mixtures [23,28], with most existing research focusing on studying the aza-PAHs separately [29,30].

The main objective of this work is to address the aforementioned gap by presenting the development and optimization of an advanced methodology based on Ultra-High-Performance Liquid Chromatography coupled with High-Resolution Mass Spectrometry (UHPLC-HRMS OrbiTrap) for the simultaneous quantitative analysis of azaarenes and nitrated and oxygenated PAHs, potentially present in fumes emitted by asphalt materials. The method’s performance was then validated and applied to the analysis of complex matrices, specifically the fumes emitted by different formulations of bituminous mixtures, to demonstrate its effectiveness.

## 2. Materials and Methods

### 2.1. Chemicals and Reagents

The method was developed using 14 analytical standards, each with a purity >98% covering the different families of PAH derivatives. The nitro-PAHs and oxy-PAHs, including 1-nitropyrene, 2-nitrofluorene, 2-naphtol, 1-hydroxypyrene, and anthraquinone, were purchased from Sigma-Aldrich and Supelco (Merck, Darmstadt, Germany). The aza-PAHs, including quinoline, carbazole, 7H-dibenzo(c,g) carbazole, dibenz(a,h)acridine, dibenz(c,h)acridine, dibenz(a,j)acridine, benz(a)acridine, benz(c)acridine, and the 6H-benzo(c,d)pyren-6-one, were purchased from LGC Standards (Teddington, Middlesex, UK) with a purity ≥ 99%. Anthraquinone d8 and quinoline d7 were used as internal standards for the negative and positive ionization mode (APCI^−^ and APCI^+^), respectively, and obtained from CIL Cluzeau (Ste. Foy-La-Grande, France) with a purity of 98%. Stock solutions of the individual PAH standards and the internal standards were prepared in dichloromethane (DCM) of HPLC-grade, purchased from Sigma-Aldrich (Merck, Darmstadt, Germany), due to the limited solubility of some compounds in acetonitrile at concentrations ranging between 0.6 and 0.2 mg mL^−1^. The solutions were stored at −18 °C in amber glass vials to prevent degradation. These stock solutions were used to prepare diluted working mixtures in acetonitrile (ACN), UHPLC-MS Optima purchased from Fisher Scientific SAS (Illkirch, France). These mixtures were used to prepare calibration standards in (ACN/H2O) (50/50, *v*/*v*) to ensure compatibility with the LC-MS.

### 2.2. LC-MS Conditions and Analysis Parameters

The LC-MS analysis was conducted using an Orbitrap Eclipse mass spectrometer coupled to a Vanquish Flex Quaternary Ultra-High-Performance Liquid Chromatography (UHPLC) system (Thermo Fisher Scientific, San Jose, CA, USA).

Prior to developing the method, each compound was infused directly into the mass spectrometer at a concentration of 1 mg L^−1^ using a syringe pump at a flow rate of 5–10 µL min^−1^. Both Atmospheric-Pressure Chemical Ionization (APCI) and ElectroSpray Ionization (ESI) were tested in positive and negative modes to determine the most efficient ionization source. While ESI was tested, several analytes showed weak or no response under both polarities. In contrast, APCI provided consistent and higher signal intensities across all 14 compounds, enabling the detection of characteristic ions in the form of [M + H]^+^, [M − H]^−^, and [M]^•−^. Based on these tests, APCI was selected as the optimal choice for both positive and negative ionization modes, ensuring proper ionization and response for all compounds. The corresponding mass spectra for each compound are provided in Figures S1–S14 of the Supplementary Materials.

A Hypersil Gold C18 column (100 mm × 2.1 mm, 1.9 µm) maintained at 30 °C was used to separate the different analytes. The mobile phase consisted of water (A) and acetonitrile (B). Chromatographic separation was carried out using a gradient elution over a total run time of 12 min at a constant flow rate of 0.5 mL min^−1^ and an injection volume of 3 µL. The full gradient is displayed in Table 1.

Instrumental parameters were first optimized individually for each compound by direct infusion. The final selected conditions represented a compromise that ensured adequate ionization efficiency and satisfactory signal intensity across all analytes. Specifically, the APCI source parameters, such as the sheath gas and auxiliary flow rate, were set at 45 and 5 (arbitrary units), respectively, to obtain better signal intensity. In addition, positive and negative discharge currents were applied at 4 and 10 µA, respectively, and the vaporizer and the ion transfer tube temperatures were set at 350 and 300 °C, respectively. Furthermore, the distance and the axial position of the APCI probe were optimized, with the settings adjusted to 2 and Medium, respectively, to enhance signal intensity, ion transmission, and overall ionization efficiency. The RF lens was set at 60%, ensuring an equilibrium between sensitivity and ion transmission efficiency. Full scan mode was applied with a mass-to-charge (*m*/*z*) range of 80 to 1200 and a resolution of 60,000 FWHM. A summary of the tested and optimized instrumental parameters for both the UHPLC and APCI-MS settings is provided in Table 2.

### 2.3. Quantification, Calibration, and Sensitivity Assessment

In accordance with ICH Q2(R2) guidelines [31], the method developed in this study was rigorously validated to ensure its effectiveness. This involved assessing the method’s linearity, accuracy, and intra- and inter-day precision, as well as determining the limits of detection (LODs) and quantification (LOQs).

The limits of detection (LODs) and quantification (LOQs) for each analyte were assessed by injecting triplicate samples of standard solutions containing each targeted PAH derivative at different concentrations. These values were calculated based on the signal-to-noise ratio (S/N) with

$$LOD=3.3\times \left(\frac{\sigma }{S}\right)\mathrm{and}\;LOQ=10\times \left(\frac{\sigma }{S}\right)$$

where **σ** is the standard deviation of the y-intercept of the regression lines and S is the slope of the calibration curve.

### 2.4. Sample Collection, Preparation, and Extraction

An experiment was conducted at the MIT laboratory, Gustave Eiffel University, Nantes, France, where a prototype simulating real-world asphalt production was operated to generate fumes from different bituminous mix designs [32]. These fumes were collected on 47 mm diameter quartz filters (Tissuquartz 2500 QAT-UP, Pall Corporation, Port Washington, NY, USA), which were pre-conditioned at 500 °C for 4 h before sampling. The flow of 4.8 L min^−1^ for 1 h. A clean air dilution system was applied during sampling to prevent filter saturation.

Prior to the analysis of these filters, an extraction procedure was conducted using a Dionex ASE 350 Accelerated Solvent Extraction device (Thermo Fisher Scientific, San Jose, CA, USA) and validated using standard solutions by spiking clean filters with a mixture of targeted PAH derivatives and their internal standards, which were then placed in a 33 mL stainless steel cell filled with glass beads. After optimizing the number of extraction cycles (2, 3, and 4 were tested on the deuterated standards shown in Table S1 in SI), these filters underwent a 3-cycle extraction with dichloromethane and acetone (50/50, *v*/*v*) at 100 °C under a pressure of 100 bars, with a heat-up time of 5 min, a static time of 5 min, a purge time of 100 s and a flush volume of 60% of the extraction cell volume. Once extraction was complete, the extracts were concentrated by reducing the volume to 10 mL under a nitrogen stream at 40 °C using an evaporation unit (TurboVap II, Biotage, Uppsala, Sweden).

Following the validation of the optimized ASE method, the collected filters were extracted using the same process and concentrated to a final volume of 500 µL to ensure optimal sensitivity and compatibility with LC-MS-Orbitrap analysis. No further cleanup step was applied prior to the injection. To prevent potential carryover, blank injections were systematically performed between runs.

After sample injection, the concentrations of target compounds in the filter extracts were determined using external calibration curves. These curves were generated by plotting the peak area ratio of each analyte to its corresponding internal standard across 5 to 9 concentration levels, depending on the compound (see Section 3.1.1). A simple linear regression was applied (y=ax+b) using Microsoft Excel, and each calibration level was injected in triplicate to ensure reproducibility. All data acquisition, processing, and quantification were performed using Thermo Scientific XCalibur Software (version 4.5.474.0) and Excel.

## 3. Results and Discussion

### 3.1. Method Validation

#### 3.1.1. Linearity, Accuracy, and Sensitivity

Linearity was evaluated based on the calibration curves described in the previous section. A strong coefficient of determination, R^2^ > 0.99, was consistently obtained for each PAH derivative, indicating an adequate linearity for the tested ranges.

Following the evaluation of linearity, accuracy was assessed through the infusion experiments as described in Section 2.2. This allowed the determination of the exact mass of the detected ions, including [M + H]^+^, [M − H]^−^, and [M]^•−^ ions. The high resolution of the mass spectrometer ensured accurate mass measurements with a mass accuracy ranging between 0.2 and 3 ppm, thereby reducing the probability of inaccurate identifications caused by isobaric interferences. The retention time of each analyte was also optimized by injecting a mixture of standard solutions containing all the studied compounds into the LC-MS system, thereby avoiding coelution between targeted PAH isomers and ensuring better chromatographic selectivity.

While most of the analyzed compounds exhibited lower LOD and LOQ values in APCI^+^ ionization mode, those identified in APCI^−^ mode had slightly higher LOD and LOQ values due to lower ionization efficiency in negative mode, resulting in reduced sensitivity. Nevertheless, the method provided sufficient sensitivity for the accurate analysis of PAH derivatives at trace levels across both ionization modes.

All evaluated validation parameters discussed below, such as the retention time, exact mass, R^2^, LOD, and LOQ, as well as the calibration range for each compound, are summarized in Table 3.

The method developed in this study for analyzing PAH derivatives (oxy-, nitro- and aza-PAHs) was more sensitive than previously reported methods. For instance, a previous study developed for the analysis of oxy-, nitro-, and nitrogen-containing PAHs using an LC-MS-APCI [15], reported higher limits of detection and quantification ranging from 0.1 to 18.5 µg L^−1^ and 3 to 61.6 µg L^−1^, respectively. In contrast, the present method achieved more consistent LODs and LOQs with significantly lower values ranging from 0.12 to 0.61 µg L^−1^ and from 0.36 to 1.85 µg L^−1^, respectively. Another study, by O’Connell et al. [10], using GC-EI-MS and LC-APCI-MS, also reported higher LODs and LOQs for two common oxy-PAHs analyzed in our study: anthraquinone and 6H-benzo(c,d) pyrene-6-one. Specifically, the LODs for 6H-Benzo(c,d) pyrene-6-one were 1.1 µg L ^−1^ (GC-EI-MS) and 2.6 µg L^−1^ (LC-MS-APCI) while the LOQs were 5.5 and 13 µg L^−1^, respectively. For anthraquinone, the LODs were 6.9 µg L^−1^ (GC-EI-MS) and 3.6 µg L^−1^ (LC-MS-APCI) with corresponding LOQs of 35 and 18 µg L^−1^. By contrast, our study, which used LC-APCI-HRMS, reported lower LODs and LOQs for both compounds, with LODs of 0.15 and 0.28 µg L^−1^ and LOQs of 0.45 and 0.85 µg L^−1^, respectively.

A more recent study, using GC-MS/MS, reported higher LODs and LOQs for three compounds, also analyzed in our study: 2-nitrofluorene with a LOD of 1.3 µg L^−1^ and a LOQ of 6.7 µg L^−1^; 1-nitropyrene with a LOD of 4 µg L^−1^ and a LOQ of 20 µg L^−1^, and 7H-dibenzo(c,g)carbazole with a LOD of 0.15 µg L^−1^ and a LOQ of 0.7 µg L^−1^ [33]. By contrast, our method achieved significantly lower LODs and LOQs for the first two compounds (LODs: 0.31 and 0.27 µg L^−1^ and LOQs: 0.95 and 0.85 µg L^−1^, respectively) and a comparable LOD and improved LOQ for 7H-dibenzo(c,g)carbazole (0.15 and 0.48 µg L^−1^).

This method has therefore been shown to be highly sensitive, achieving low LOD and LOQ values, making it suitable for identifying and quantifying PAH derivatives at ultra-trace levels.

#### 3.1.2. Precision, Repeatability, and Reproducibility

The reliability of the developed method was assessed by evaluating intra- and inter-day precision. Analytical standards were prepared by diluting stock solutions at the different concentrations along the calibration curve. These were analyzed three times within a single day and over three consecutive days while staying in the autosampler at −10 °C to determine the repeatability and reproducibility, respectively, by calculating the coefficient of variation (% RSD). The % RSD value for each compound was averaged across all concentrations to provide a single % RSD per day and simplify the data representation. The overall % RSD was calculated for reproducibility across all three days. The different % RSD values representing repeatability (intra-day precision) and reproducibility (inter-day precision) are summarized in Table 4.

As shown in Table 4, satisfactory precision was achieved with all compounds, as demonstrated by the low % RSD values that fell within the acceptable limit (i.e., <20%) for analytical methods. While compounds analyzed in positive ionization mode (APCI^+^) showed excellent repeatability, with % RSD values varying between 3 and 6%, and a great reproducibility, with % RSD values ranging between 3 and 9%, compounds ionized in negative mode (APCI^−^) demonstrated a slightly higher % RSD values varying between 4 and 12% for repeatability and between 8 and 12% for reproducibility, indicating higher variability across days. Despite these variations, analysis in both modes remained highly precise (<15%), confirming the method’s efficiency in accurately quantifying PAH derivatives.

#### 3.1.3. Matrix Effect Evaluation

**Figure 1:**
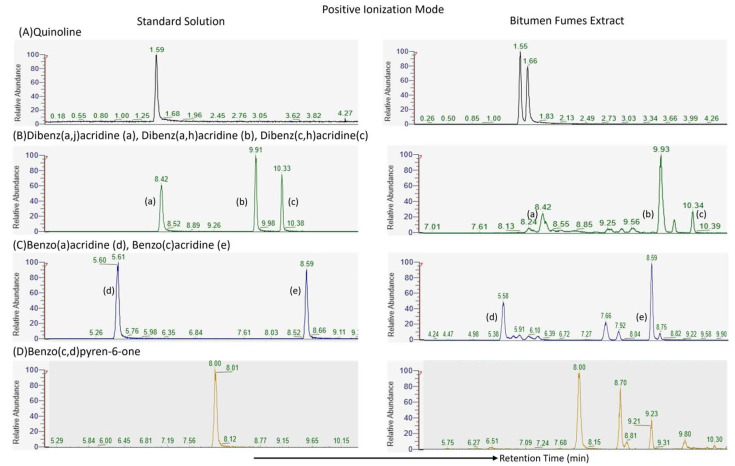
Extracted ion chromatograms (EICs) of target compounds acquired in positive ion mode. Each panel displays a comparison between a standard solution (right column) and a bitumen fume extract (left column).

**Figure 2:**
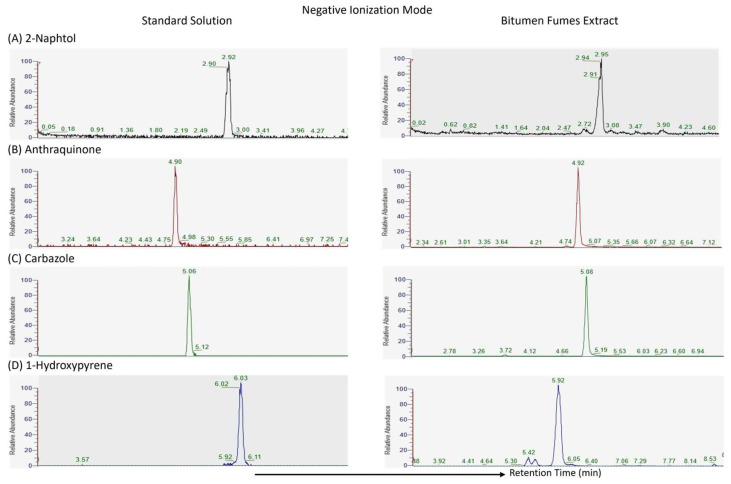
Extracted ion chromatograms (EICs) of target compounds acquired in negative ion mode. Each panel displays a comparison between a standard solution (right column) and a bitumen fume extract (left column).

Although the matrix effect was not quantified using a dedicated post-column infusion or spiking approach, a qualitative assessment was performed to evaluate potential interferences caused by the complex fume matrix. Extracted ion chromatograms (EICs) were compared between standard solutions and bitumen fume extracts for all target compounds in both positive and negative ion modes (Figure 1 and Figure 2).

These chromatograms showed consistent retention times and peak shapes across matrices, with no signs of overlapping peaks or increased baseline noise, indicating the absence of significant coeluting matrix interferences. Furthermore, the high resolution of the Orbitrap mass spectrometry enhanced selectivity, allowing the precise identification and quantification of all targeted compounds.

This qualitative comparison confirmed that the developed method was robust and selective, even without a dedicated cleanup step.

To complement this visual comparison, a quantitative estimation of potential background interference was performed using the following equation:

$$\frac{{(Noise\;level}_{Sample}-{Noise\;level}_{Standard})}{{Max\;peak\;height}_{sample}}\times 100$$

Here, “Noise level” corresponds to the mean signal intensity in a retention-time window (±2 min) around the analyte peak, excluding the peak itself. For compounds absent in the sample, the calculation was reported as not applicable (NA). Negative values reflect a slight matrix-induced signal enhancement; however, in this study, all such values were below 10% and therefore considered negligible. The results of this estimation are presented in Table 3, confirming that the impact of matrix-induced background noise was generally minimal for the investigated compounds.

### 3.2. Application to Fume Samples from Bituminous Pavement Materials

Prior to analyzing fume samples from bituminous mixtures, recovery rates were evaluated using standard solutions to ensure the effectiveness of the extraction method described in Section 2.4.

As shown in Table 5, the recovery rates of most compounds fell within the acceptable range for trace-level environmental analysis, ranging from 67% to 109%. However, compounds analyzed in APCI positive mode using quinoline-d7 as an internal standard consistently showed recovery rates exceeding 100%, with quinoline averaging 129% across multiple independent triplicates. This overestimation was unlikely to be related to the internal standard (quinoline-d7) as quinoline and its deuterated analogue share identical structural and physicochemical properties. If evaporation losses had occurred, both compounds would have been affected similarly, resulting in a recovery closer to 100%. The observed value (129.6%) was, therefore, more likely due to minor procedural uncertainties, especially at low concentration levels.

Conversely, compounds analyzed in APCI negative mode used anthraquinone-d8, a less volatile internal standard, which remained more stable during the evaporation process. In this case, the stronger internal standard signal may have led to a slight underestimation of the analyte response, resulting in systematically lower recoveries (<100%).

Acceptable% RSD values below 20% were observed for most compounds, except for quinoline (21.9%), 1-hydroxypyrene (39%), and 2-nitrofluorene (29%). These higher RSD values were consistent across multiple replicates and were likely attributed to losses during the evaporation step, likely related to their high volatility. This was further supported by the good reproducibility observed for these compounds during method validation with %RSD values < 15% (see Section 3.1.2), confirming the compounds’ stability during analysis. Overall, these variations reflected compound-specific behavior rather than random experimental variability.

Nevertheless, the method demonstrated consistent, effective, and suitable extraction performance for the targeted PAH derivatives.

**Figure 3:**
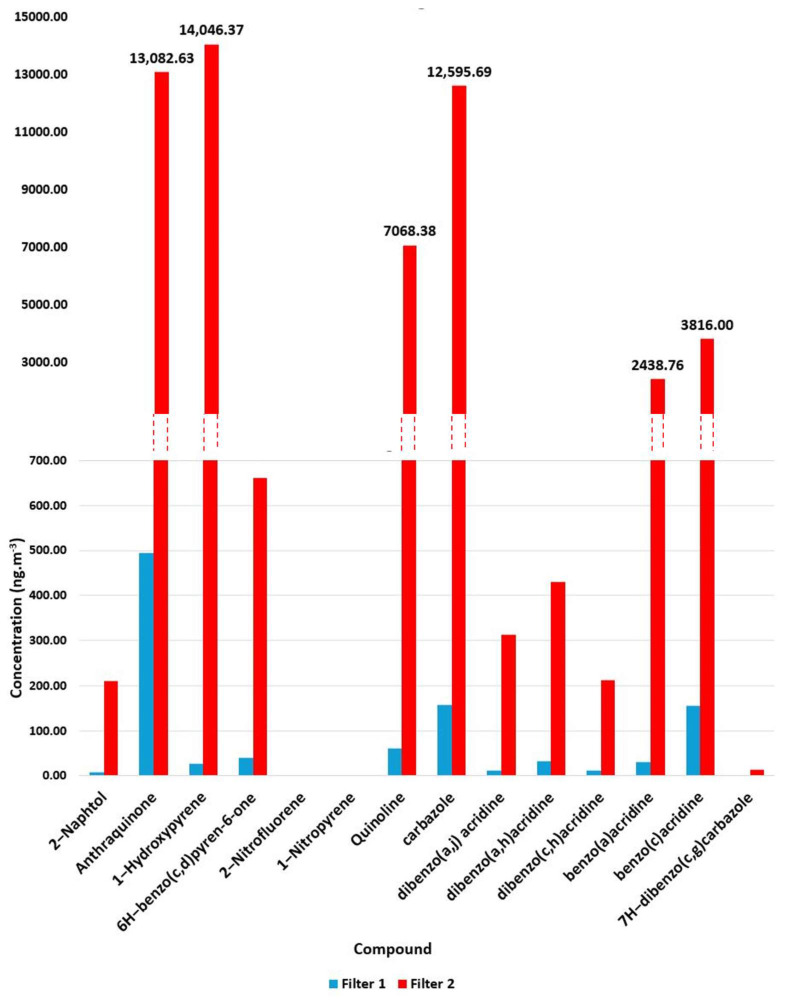
Concentration of the PAH derivatives identified in emissions from two different bituminous mixtures. Filter 1 corresponds to a mix design of 35/50 pen-grade bitumen and virgin aggregates. Filter 2 corresponds to a mix design with 70/100 pen-grade bitumen with a 50:50 blend of virgin and reclaimed aggregates.

After validating the extraction process, the results of two bitumen fume samples will be presented to demonstrate the applicability of the method. Following “low volume” sampling (0.288 m^3^ h^−1^) of emitted fumes, quartz filters were extracted in accordance with the methodology described in Section 2.4. As shown in Figure 3, the analysis of these samples enabled the identification and quantification of all targeted PAH derivatives. Air emissions from pavement materials used for the wearing course of highly trafficked roads were studied during the manufacturing and mixing process at 160 °C. Filter 1 corresponded to fumes emitted from a mix design formulated with 35/50 pen-grade bitumen and virgin aggregates, both of which were heated at 160 °C. Filter 2 was used to collect fumes from a second mix design incorporating 70/100 pen-grade bitumen and a blend of aggregates consisting of 50% (*w*/*w*) virgin aggregates (identical to those used in Filter 1) and 50% (*w*/*w*) reclaimed asphalt pavement (RAP), which was also heated at 160 °C. The RAP originated from the recycling of an old French road contaminated by tar or pitch. It included two types of material: one highly polluted (high level of PAH) and one considered non-polluted. The detailed results are presented in Table S2 of the Supplementary Materials.

As shown in Figure 3, marked differences were observed in the emission profiles of the two asphalt mixtures. For instance, the total PAH derivatives load rose from 1049 ng m^−3^ for Filter 1 to almost 54,891 ng m^−3^ for Filter 2 (fumes from the sample containing polluted reclaimed asphalt pavement).

Certain compounds, such as 1-hydroxypyrene, quinoline, and carbazole, exhibited notable increases in concentrations between Filters 1 and 2 by factors of 511, 117, and 80, respectively. Other compounds, such as dibenzo(a,h) acridine (×12) and 2-naphthol (×24), exhibited much lower increase factors. These variations may have reflected formulation-dependent differences or operating conditions, but given the limited sample set, no firm conclusions can be drawn. The observed differences served to illustrate the method’s ability to detect and quantify a wide range of concentrations across various PAH derivative families, including azaarenes, nitro-, and oxy-PAHs, while maintaining compound-specific resolution. This reflects the method’s selectivity, sensitivity, and adaptability to different formulations and complex matrices, making it suitable for analyzing industrial and environmental samples.

It is important to note, however, that the results presented in this study were based on only two fume samples, each of which originated from a different bituminous mixture used as a wearing course and composed of different aggregates (virgin and recycled) and binders. These mixtures were produced under distinctive operating conditions. Consequently, the findings should be interpreted only as an application of the analytical method, not as part of a comparative emission study. Further work involving a broader range of bituminous mixtures and controlled experimental conditions is required to assess whether the observed emission patterns apply more broadly across different bitumen types.

## 4. Conclusions

The methodology developed in this study demonstrated high sensitivity, mass accuracy, and precision. It enabled the simultaneous analysis of 14 different PAH derivatives, including four oxy-PAHs, two nitro-PAHs, and eight aza-PAHs. The low LODs and LOQs achieved in this study confirm the method’s suitability for trace-level analysis. Its successful application to the analysis of fumes emitted by bituminous mixtures highlighted its ability to analyze complex matrices without the need for a cleanup step, overcoming a major limitation of conventional methods. All 14 targeted compounds were identified and quantified in two distinct fume samples of bituminous mixtures, which exhibited different emission profiles with dynamic variability in derivative concentrations. This confirms the robustness and applicability of the method to various complex environmental and industrial matrices, offering a reliable, efficient, and sensitive tool for the simultaneous detection and quantification of different PAH derivatives. This advancement supports enhanced regulatory compliance and enables a more precise assessment of risks and exposure for both workers and the environment. In future developments, efforts will focus on exploring greener extraction techniques such as those with supercritical CO_2_ to reduce solvent usage and improve the overall sustainability of the method.

## Figures and Tables

**Table 1:** Elution gradient of the mobile phase.

Step	Time (min)	% A	% B	Comments
Initial Conditions	0	70	30	Start
Linear Gradient to 50% B	0–4	50	50	Linearly increase B
Hold at 50% B	4–6	50	50	Isocratic
Linear Gradient to 95% B	6–10	5	95	Linearly increase B
Hold at 95% B	10–11	5	95	Strong elution phase
Re-equilibration	11–12	70	30	Return to initial conditions

**Table 2:** Summary of instrumental parameters tested and selected for UHPLC and APCI-MS optimization. The optimal values represent a compromise that ensured satisfactory response for all 14 PAH derivatives.

Parameter	Optimized Value
**Liquid Chromatography (UHPLC)**	
Column Length C_18_ (5 cm and 10 cm)	C_18_, 100 × 2.1 mm, 1.9 µm (Hypersil GOLD)
Mobile Phase A	100% H_2_O
Mobile Phase B	100% Acetonitrile
Gradient Program (optimized)	30% B (start), ramp to 50% in 4 min → hold for 2 min → ramp to 95% in 4 min → hold for 1 min → re-equilibration for 1 min.
Flow	0.5 mL min^−1^
Column Temperature	30 °C
Injection Volume (1, 2, 3, 5 µL)	3 µL
Time	12 min
**Mass Spectrometry (HRMS)**	
Ionization Mode (+/−)	APCI (+/−) depending on the compound
Vaporizer Temperature (between 250 and 400 °C)	350 °C
Ion Transfer Tube Temperature (200 to 300 °C)	300 °C
Sheath Gas (20–45)	45 (arbitrary units)
Auxiliary Flow Rate	5 (arbitrary units)
Positive Discharge Current	4.0 µA
Negative Discharge Current	10 µA [10,15]
RF Lens (30 and 60%)	60%
Axial Position of the APCI-Probe (High and Medium)	Medium
Distance of the APCI-Probe (3 and 2)	2
Full Scan Range	*m*/*z* 80–1200
Resolution	60,000 FWHM

**Table 3:** Summary of linearity, sensitivity, and accuracy for PAH derivatives.

PAH Derivative	Retention Time (min)	Exact Mass (*m*/*z*)	Calibration Range	Linearity R^2^	LOD (µg L^−1^)	LOQ (µg L^−1^)	Baseline Noise Impact (%)
Quinoline *	1.56–1.59	130.0652 [M + H]^+^	0.5–15	0.9987	0.12	0.36	−4
2-Naphtol ^†^	2.91–2.95	143.0503 [M − H]^−^	1–15	0.9957	0.25	0.76	2
Anthraquinone ^†^	4.90–4.94	208.0529 [M]^•−^	1–15	0.9985	0.28	0.85	−8
Carbazole ^†^	5.05–5.09	166.0662 [M − H]^−^	1–15	0.9929	0.28	0.85	0
Benzo(a)acridine *	5.57–5.61	230.0976 [M + H]^+^	0.5–15	0.9985	0.11	0.35	5
1-Hydroxypyrene ^†^	6.03–6.08	217.0657 [M − H]^−^	2–15	0.9988	0.61	1.87	0
2-Nitrofluorene ^†^	7.06–7.11	210.0560 [M − H]^−^	1–15	0.9918	0.31	0.95	NA
6H-benzo(c,d)pyren-6-one *	7.97–8.00	255.0803 [M + H]^+^	0.5–15	0.9991	0.15	0.45	0
Dibenz(a,j)acridine *	8.40–8.43	280.1121 [M + H]^+^	1–15	0.9985	0.22	0.68	2
Benzo(c)acridine *	8.57–8.59	230.0977 [M + H]^+^	0.3–15	0.9997	0.08	0.26	3
1-Nitropyrene ^†^	8.59–8.61	247.0636 [M]^•−^	1–15	0.9984	0.27	0.85	NA
7H-dibenzo(c,g)carbazole ^†^	8.75–8.78	266.0983 [M − H]^−^	0.5–15	0.9981	0.15	0.48	0
Dibenz(a,h)acridine *	9.90–9.92	280.1121 [M + H]^+^	0.5–15	0.9993	0.14	0.43	3
Dibenz(c,h)acridine *	10.32–10.34	280.1122 [M + H]^+^	0.5–15	0.9997	0.1	0.33	3

* Corrected using quinoline-d7 as internal standard; ^†^ Corrected using anthraquinone-d8 as internal standard; NA: compound absent in the sample; calculation not applicable.

**Table 4:** Representation of the intra- and inter-day precision for the different PAH derivatives analyzed in this study.

Compound	Repeatability (% RSD)	Reproducibility (%RSD)
Quinoline	5.2	6.2
2-Naphtol	7.4	10.1
Anthraquinone	8.2	9.1
Carbazole	6.8	8.6
1-Hydroxypyrene	6.9	12.2
2-Nitrofluorene	9.1	10.4
1-Nitropyrene	9.9	10.2
Dibenzo(a,j)acridine	3.4	4.1
Dibenzo(a,h)acridine	3.8	4.3
Dibenzo(c,h)acridine	3.7	4.4
Benzo(a)acridine	3.1	3.8
Benzo(c)acridine	4.0	4.1
7H-dibenzo(c,g)carbazole	5.6	10.3
6H-benzo(c,d)pyren-6-one	4.5	9.7

**Table 5:** Average recovery rates for the different PAH derivatives analyzed in this study.

Compound	Recovery Rate (%)	% RSD
Quinoline	129.6 ± 28.4	21.9
2-Naphtol	85.5 ± 5.0	5.9
Anthraquinone	101.1 ± 6.7	6.6
Carbazole	78.0 ± 9.4	12.1
1-Hydroxypyrene	74.2 ± 29	39.0
2-Nitrofluorene	67.3 ± 19.5	29.0
1-Nitropyrene	78.5 ± 11.3	14.4
Dibenzo(a,j)acridine	108.6 ± 11.6	10.7
Dibenzo(a,h)acridine	108.1 ± 10.16	9.4
Dibenzo(c,h)acridine	100.3 ± 5.6	5.6
Benzo(a)acridine	109.1 ± 9.9	9.1
Benzo(c)acridine	104.0 ± 12.0	11.5
7H-Dibenzo(c,g)carbazole	77.3 ± 14.8	19.2

## Data Availability

The original contributions presented in this study have been included in the article. Further inquiries can be directed to the corresponding author.

## Supplementary Materials

The following supporting information can be downloaded at: https://www.mdpi.com/article/10.3390/molecules30163397/s1. Figure S1: Mass Spectrum of Quinoline acquired in APCI Positive mode, showing the molecular ion [M + H]^+^ at *m*/*z* 130.0652. Figure S2: Mass Spectrum of 6H-Benzo(c,d)pyren-6-one acquired in APCI Positive mode, showing the molecular ion [M + H]^+^ at *m*/*z* 255.0803. Figure S3: Mass Spectrum of Carbazole acquired in APCI Negative mode, showing the molecular ion [M − H]^−^ at *m*/*z* 166.0662. Figure S4: Mass Spectrum of 7H-Dibenzo(c,g)carbazole acquired in APCI Negative mode, showing the molecular ion [M − H]^−^ at *m*/*z* 266.0983. Figure S5: Mass Spectrum of Anthraquinone acquired in APCI Negative mode, showing the molecular ion [M − H]^−^ at *m*/*z* 208.0529. Figure S6: Mass Spectrum of 2-Naphtol acquired in APCI Negative mode, showing the molecular ion [M − H]^−^ at *m*/*z* 143.0503. Figure S7: Mass Spectrum of 2-Nitroflurene acquired in APCI Negative mode, showing the molecular ion [M − H]^−^ at *m*/*z* 210.0560. Figure S8: Mass Spectrum of 1-Nitropyrene acquired in APCI Negative mode, showing the molecular ion [M − H]^−^ at *m*/*z* 247.0635. Figure S9: Mass Spectrum of 1-Hydroxypyrene acquired in APCI Negative mode, showing the molecular ion [M − H]^−^ at *m*/*z* 217.0657. Figure S10: Mass Spectrum of Benzo(a)acridine acquired in APCI Positive mode, showing the molecular ion [M + H]^+^ at *m*/*z* 230.0976. Figure S11: Mass Spectrum of Benzo(c)acridine acquired in APCI Positive mode, showing the molecular ion [M + H]^+^ at *m*/*z* 230.0977. Figure S12: Mass Spectrum of Dibenzo(a,j)acridine acquired in APCI Positive mode, showing the molecular ion [M + H]^+^ at *m*/*z* 280.1121. Figure S13: Mass Spectrum of Dibenzo(a,h)acridine acquired in APCI Positive mode, showing the molecular ion [M + H]^+^ at *m*/*z* 280.1121. Figure S14: Mass Spectrum of Dibenzo(c,h)acridine acquired in APCI Positive mode, showing the molecular ion [M + H]^+^ at *m*/*z* 280.1122. Table S1: Recovery rates (%) of deuterated internal standards obtained using Accelerated Solvent Extraction (ASE) with 2, 3 and 4 extraction cycles. Table S2: Detected PAH Derivatives and Their Concentrations in Two Fume Samples from Pavement Materials.

## Abbreviations

The following abbreviations have been used in this manuscript:

PAH	Polycyclic Aromatic Hydrocarbons
APCI	Atmospheric-Pressure Chemical Ionization
UHPLC	Ultra-High-Performance Liquid Chromatography
HRMS	High-Resolution Mass Spectrometry
LOD	Limit Of Detection
LOQ	Limit Of Quantification
RAP	Reclaimed Asphalt Pavement
RSD	Relative Standard Deviation
ASE	Accelerated Solvent Extraction
ESI	ElectroSpray Ionization
DCM	Dichloromethane
ACN	Acetonitrile
IARC	International Agency for Research on Cancer
WHO	World Health Organization
EPA	Environmental Protection Agency
