# Supplementary material for: Targeted LC-MS Orbitrap Method for the Analysis of Azaarenes, and Nitrated and Oxygenated PAHs in Road Paving Emissions
Source: Molecules. 2025 Aug 16;30(16):3397. doi: 10.3390/molecules30163397 (PMC12388843; doi:10.3390/molecules30163397)
Supplement: Supplementary file 1 [file molecules-30-03397-s001.zip › molecules-3795906-supplementary.pdf]

# Supplementary Information

Table S1 Recovery rates (%) of deuterated internal standards obtained using Accelerated Solvent Extraction (ASE) with 2, 3 and 4 extraction cycles.

| **Recovery Rate (%)** | **Quinoline Deuterated (m/z 137.1090)** | **Anthraquinone Deuterated (m/z 216.1032)** |
|---|---|---|
| 2 Cycles | 90.83 | 95.86 |
| 3 Cycles | 92.81 | 96.01 |
| 4 Cycles | 92.01 | 95.22 |

Table S2 Detected PAH Derivatives and Their Concentrations in Two Fume Samples from Pavement Materials.

| **Compound** | **Filter 1 (µg.L$^{-1}$)** | **Filter 1 (ng.m$^{-3}$*)** | **Filter 2 (µg.L$^{-1}$)** | **Filter 2 (ng/m$^{-3}$*)** |
|---|---|---|---|---|
| Quinoline | 3.14 | 60.51 | 217.14[a] | 7068.38 |
| 2-Naphtol | 0.45 | 8.73 | 6.49 | 211.26 |
| Anthraquinone | 25.65[a] | 494.28 | 401.90[a] | 13082.63 |
| Carbazole | 8.20 | 157.95 | 386.94[a] | 12595.69 |
| 1-Hydroxypyrene | 1.43 | 27.49 | 431.50[a] | 14046.37 |
| 2-Nitrofluorene | n.d. | n.d. | < LOQ | < LOQ |
| 1-Nitropyrene | < LOQ | < LOQ | n.d. | n.d. |
| Dibenzo (a,j) acridine | 0.61 | 11.80 | 9.62 | 313.06 |
| Dibenzo (a,h) acridine | 1.69 | 33.34 | 13.24 | 430.97 |
| Dibenzo (c,h) acridine | 0.49 | 11.00 | 6.55 | 213.15 |
| Benzo (a) acridine | 1.60 | 30.81 | 74.92[a] | 2438.76 |
| Benzo (c) acridine | 8.05 | 155.04 | 117.23[a] | 3816.00 |
| 7H-dibenzo (c,g) carbazole | < LOQ | < LOQ | 0.43 | 14.06 |
| 6H-benzo(c,d)pyren-6-one | 2.13 | 40.98 | 20.30[a] | 660.80 |

**Concentrations are corrected for the sampling dilution factor (air zero)*

*[a] A post-extraction dilution factor (×5 or ×25) was applied. Final concentrations reported here are corrected accordingly.*

*< LOQ: Detected Below the limit of Quantification*

*n.d.: Not Detected*

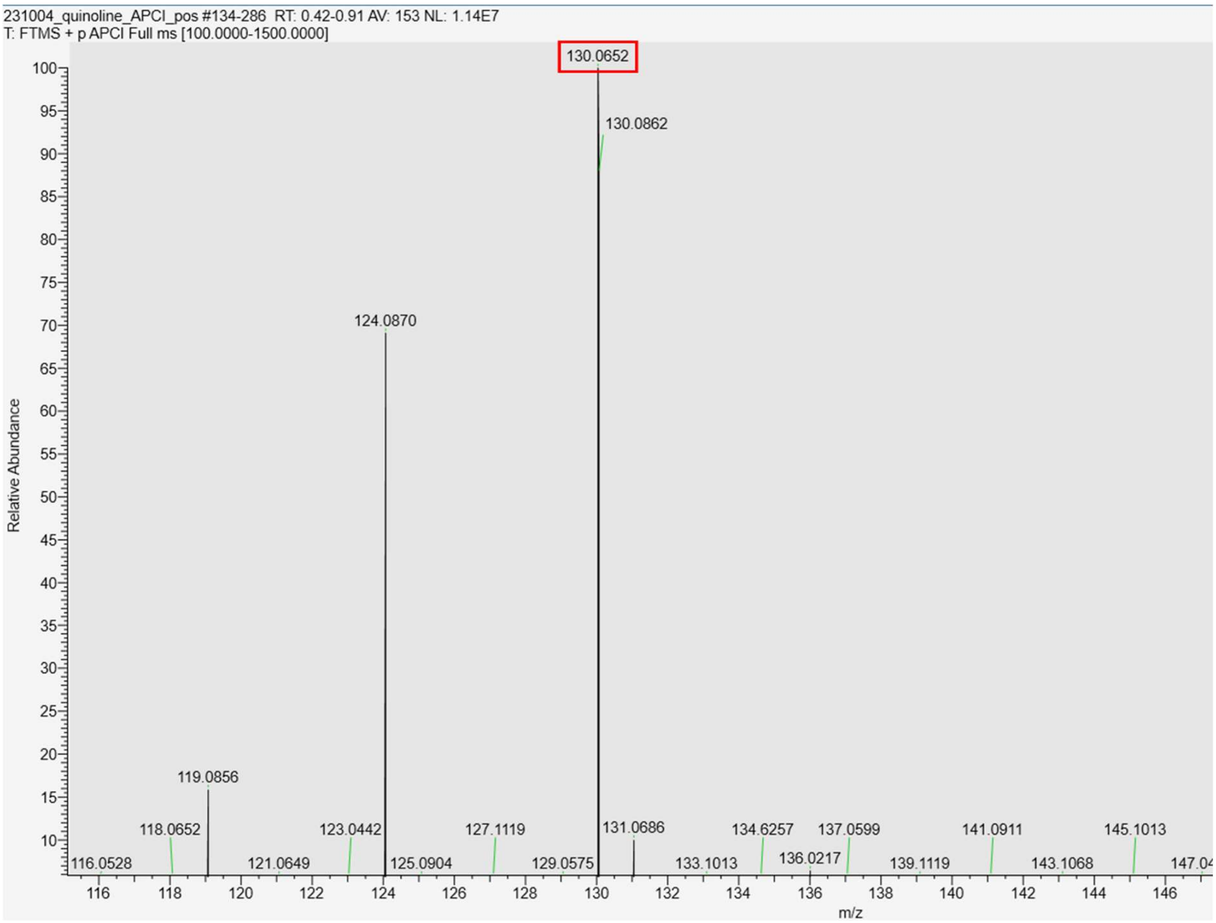


Figure S1 Mass Spectrum of Quinoline acquired in APCI Positive mode, showing the molecular ion $[M+H]^+$ at m/z 130.0652.

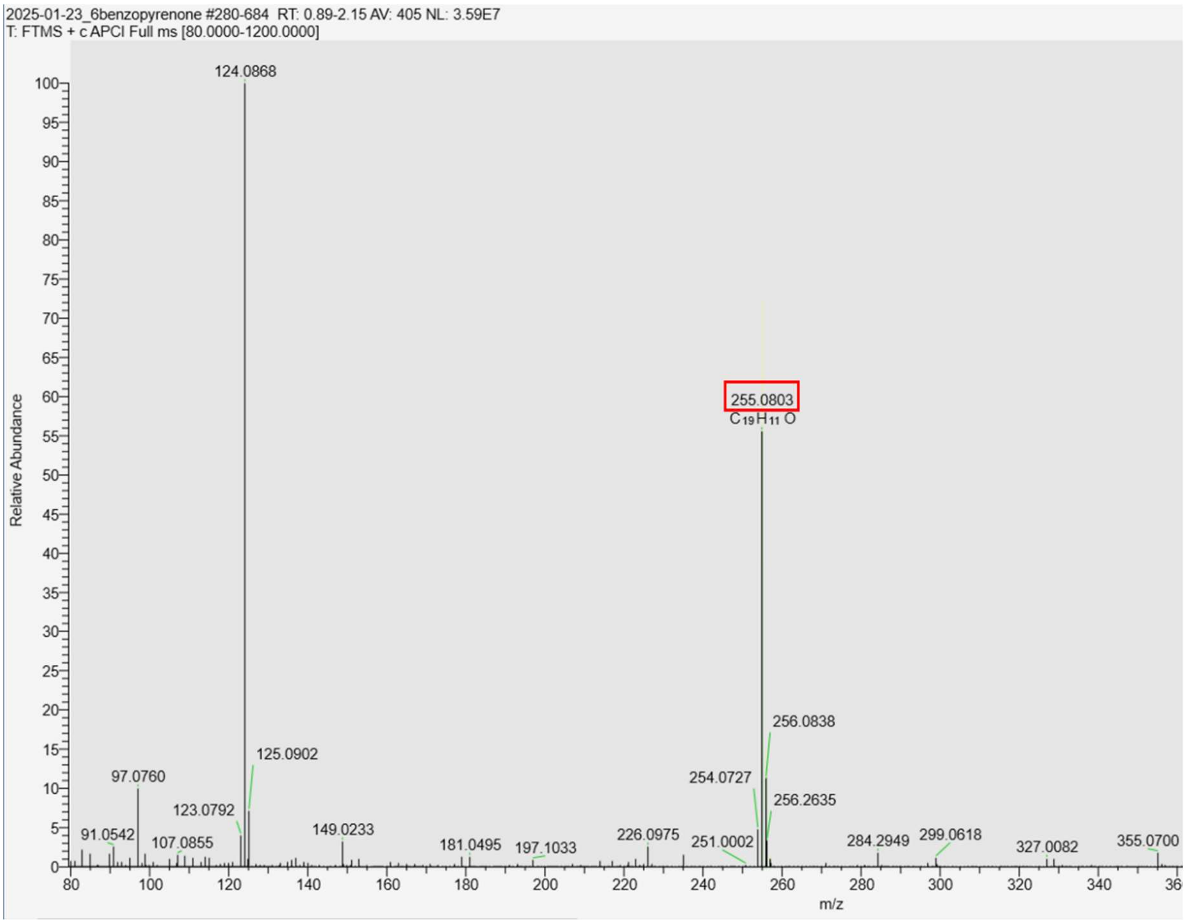


Figure S2 Mass Spectrum of 6H-Benzo(c,d)pyren-6-one acquired in APCI Positive mode, showing the molecular ion $[M+H]^+$ at m/z 255.0803.

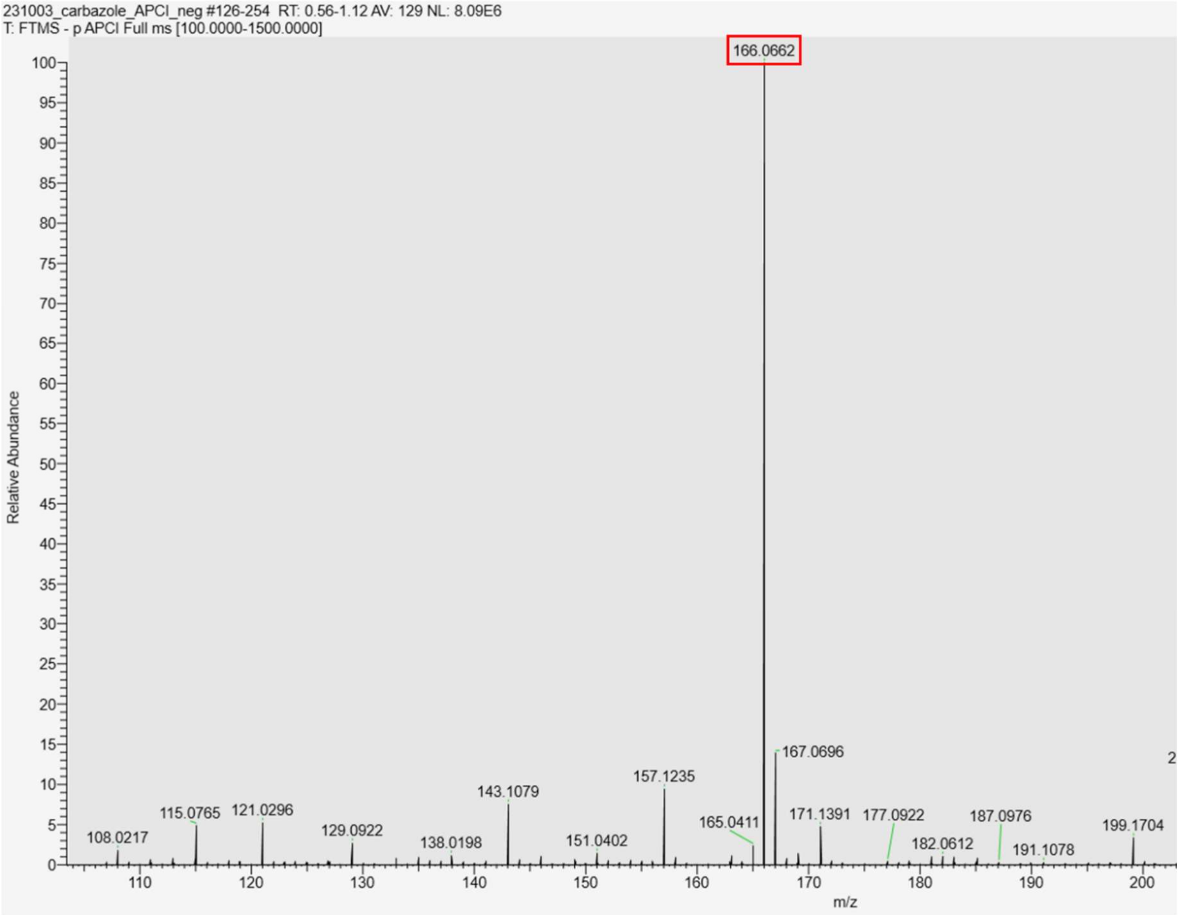


Figure S3 Mass Spectrum of Carbazole acquired in APCI Negative mode, showing the molecular ion $[M-H]^-$ at m/z 166.0662.

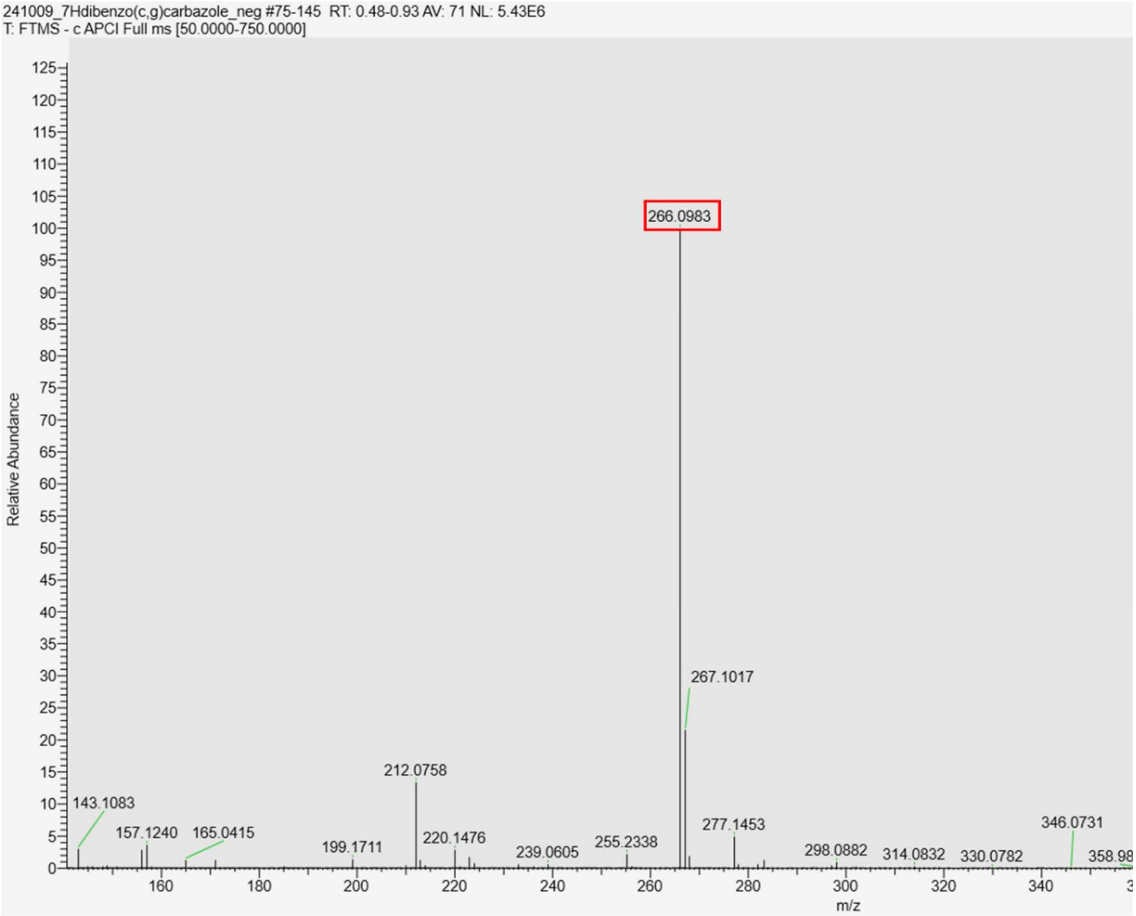


Figure S4 Mass Spectrum of 7H-Dibenzo(c,g)carbazole acquired in APCI Negative mode, showing the molecular ion [M-H]$^-$ at m/z 266.0983.

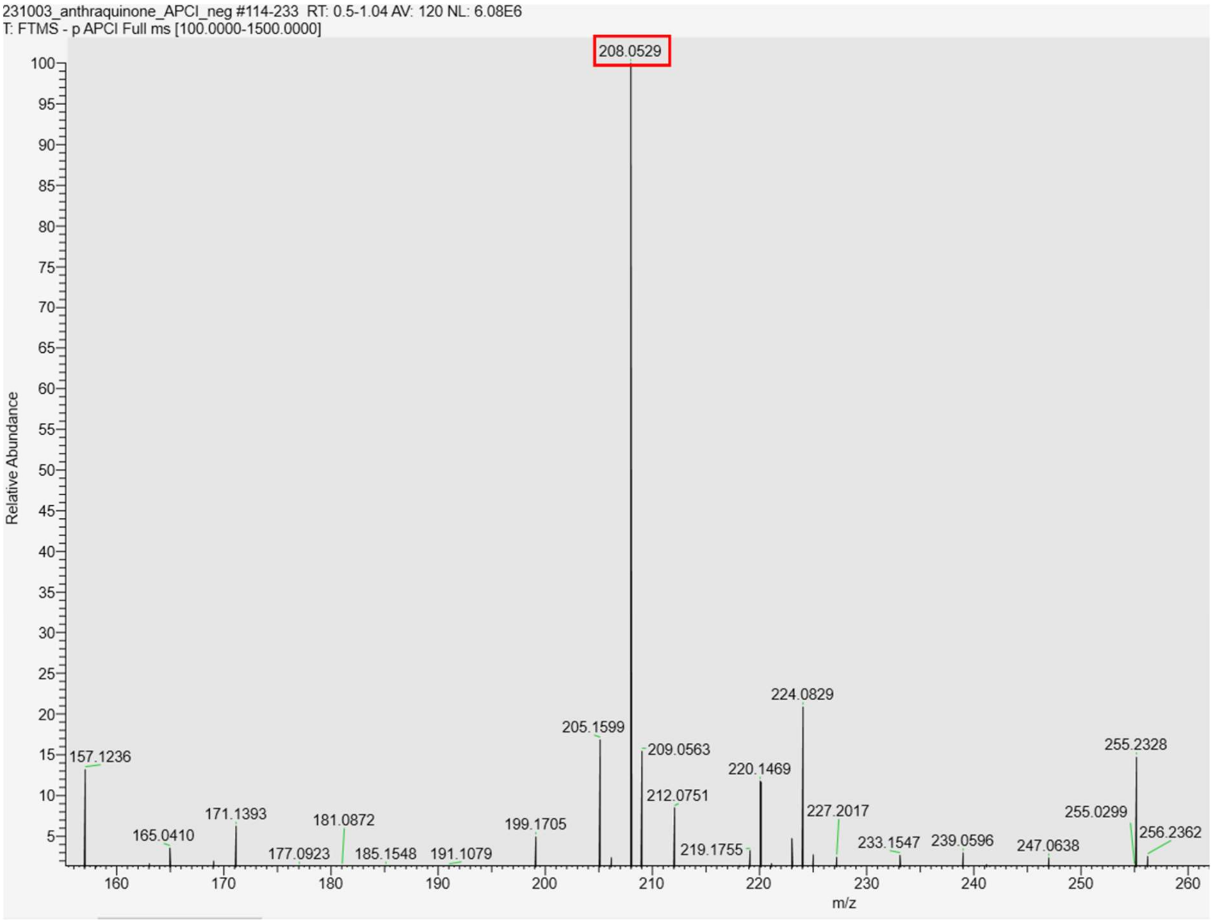


Figure S5 Mass Spectrum of Anthraquinone acquired in APCI Negative mode, showing the molecular ion $[M-H]^-$ at m/z 208.0529.

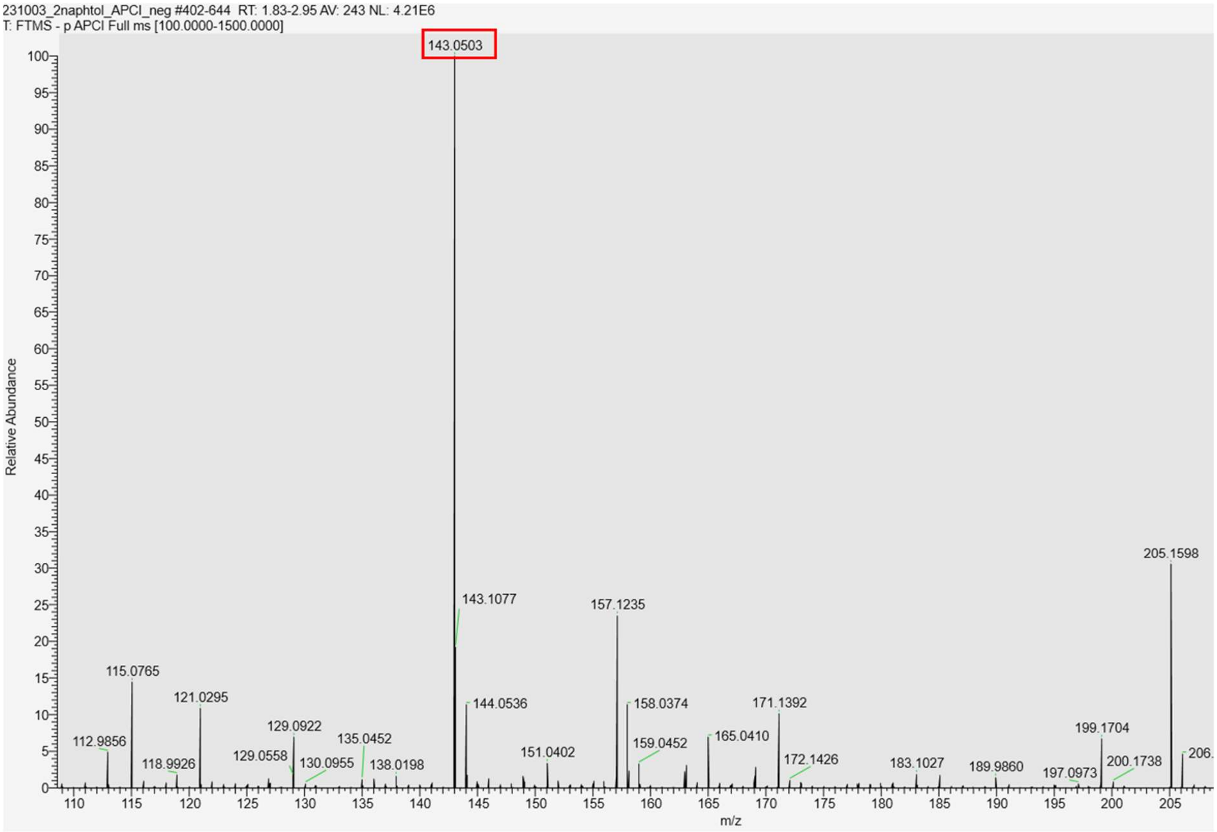


Figure S6 Mass Spectrum of 2-Naphtol acquired in APCI Negative mode, showing the molecular ion $[M-H]^-$ at m/z 143.0503.

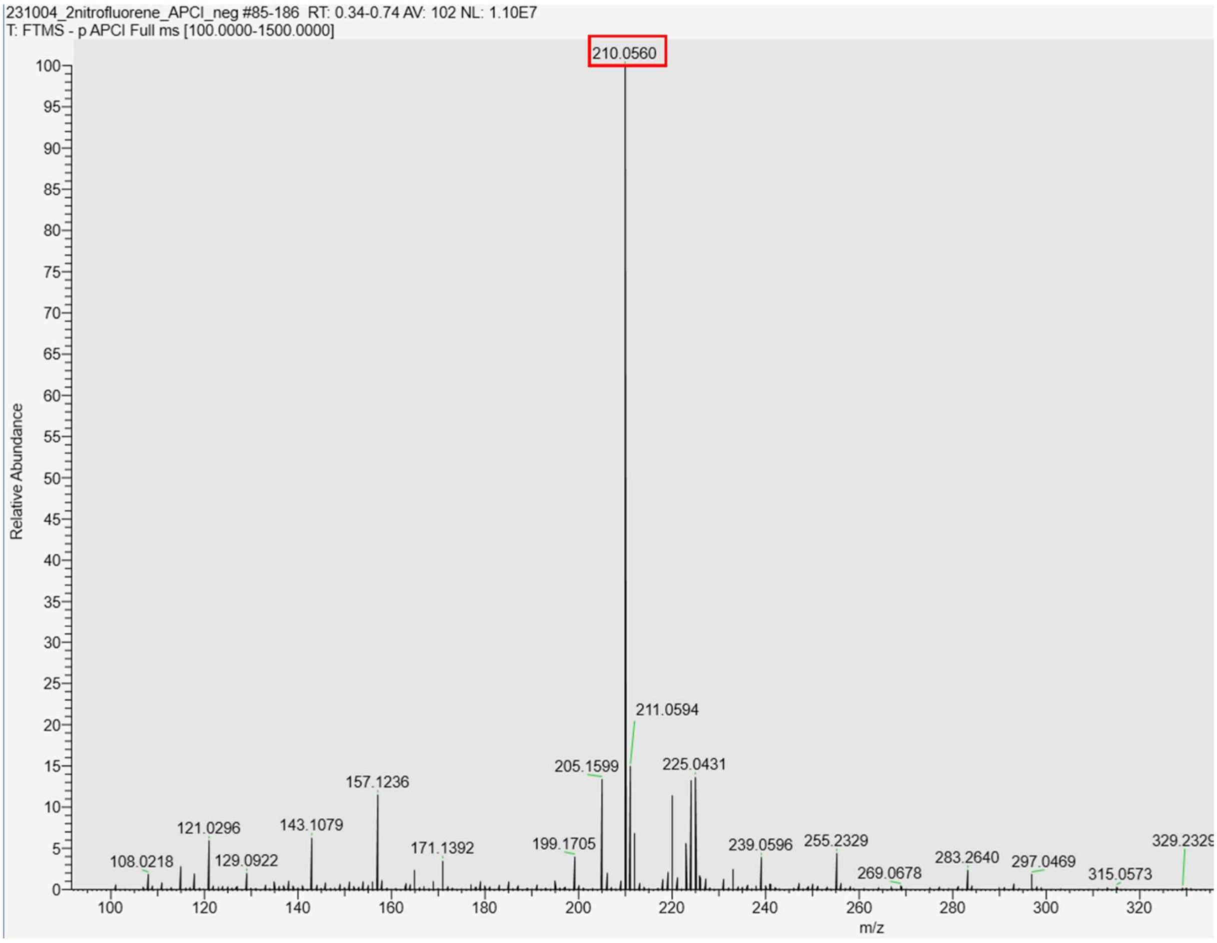


Figure S7 Mass Spectrum of 2-Nitroflurene acquired in APCI Negative mode, showing the molecular ion [M-H]$^-$ at m/z 210.0560.

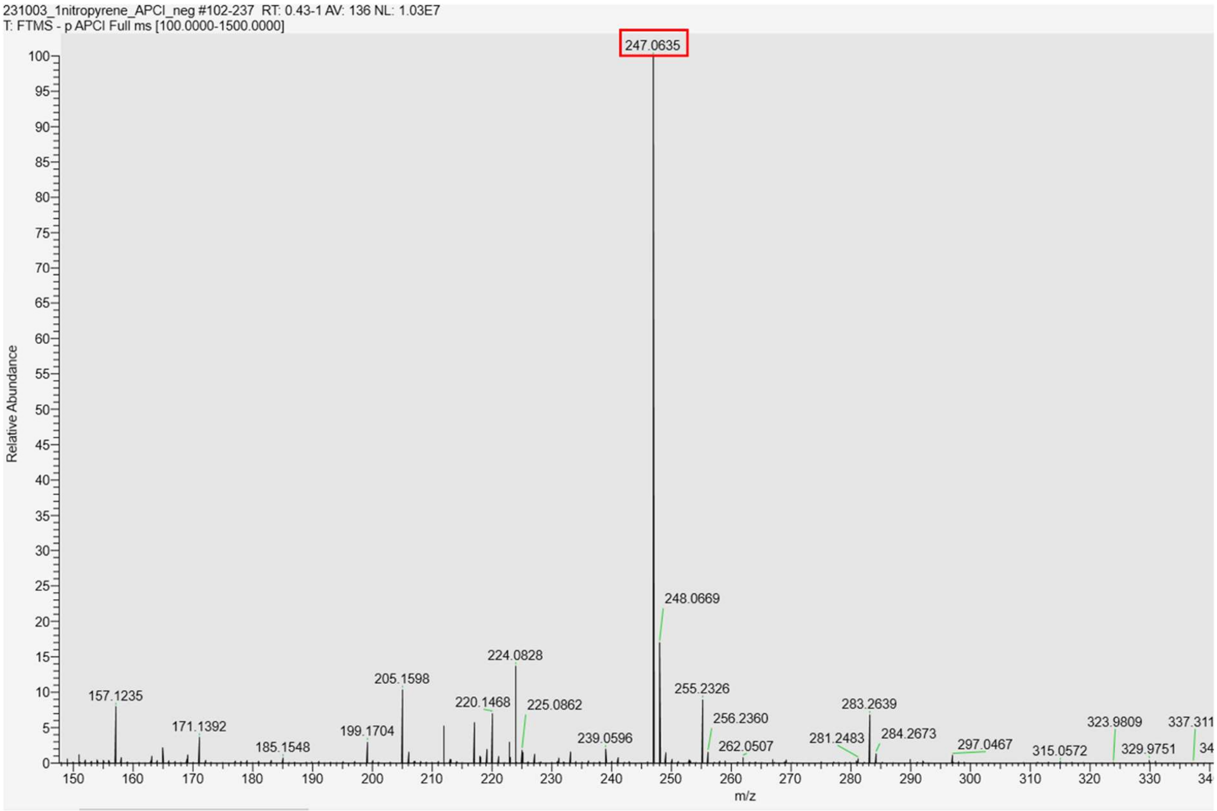


Figure S8 Mass Spectrum of 1-Nitropyrene acquired in APCI Negative mode, showing the molecular ion $[M-H]^-$ at m/z 247.0635.

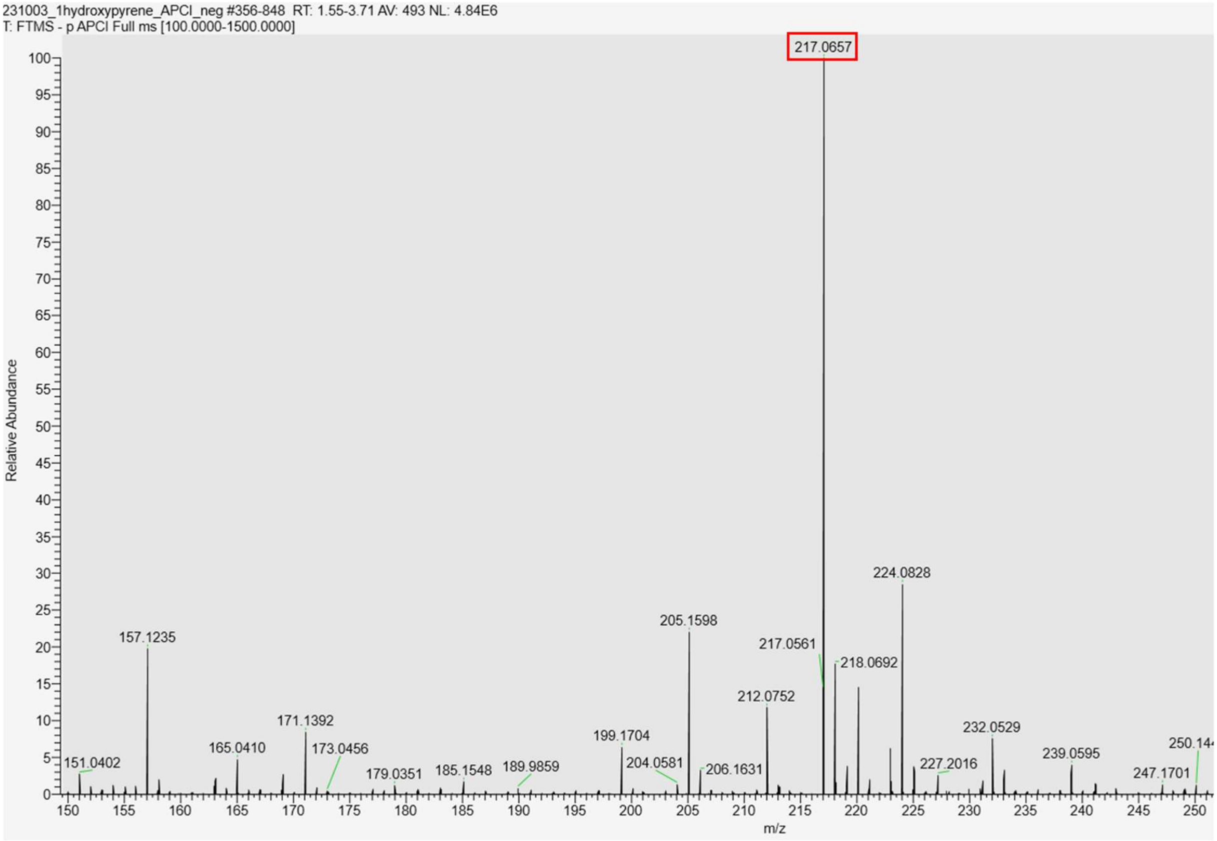


Figure S9 Mass Spectrum of 1-Hydroxypyrene acquired in APCI Negative mode, showing the molecular ion [M-H]$^-$ at m/z 217.0657.

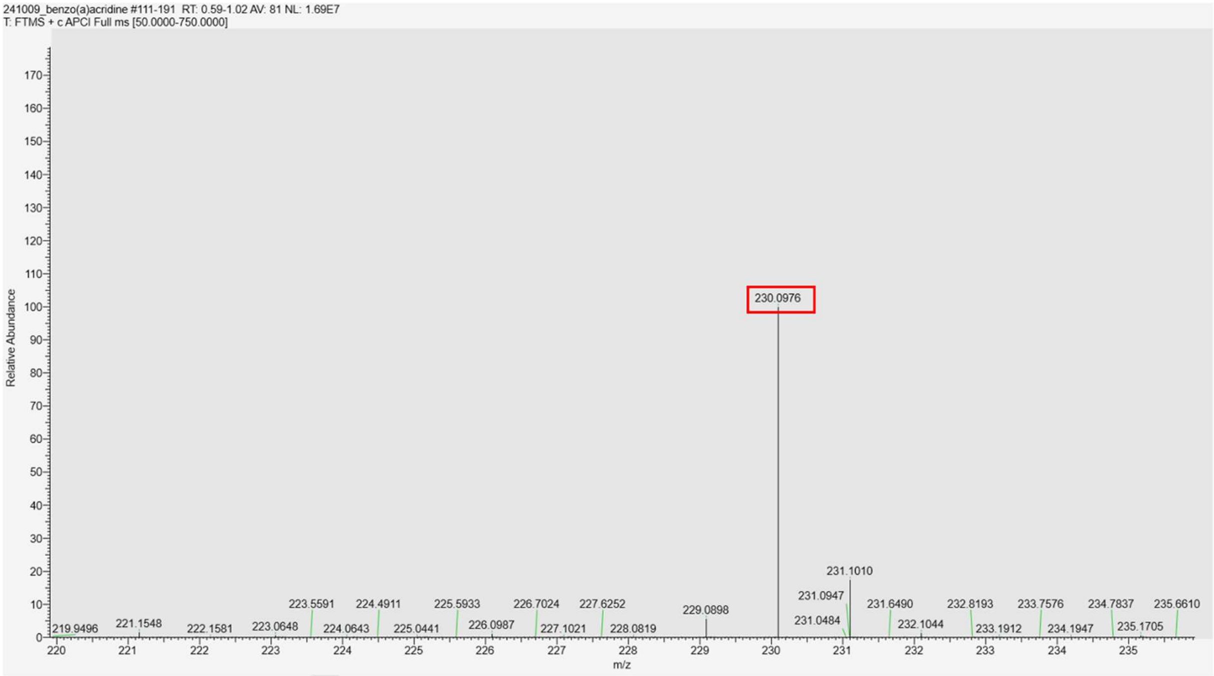


Figure S10 Mass Spectrum of Benzo(a)acridine acquired in APCI Positive mode, showing the molecular ion [M+H]$^+$ at m/z 230.0976.

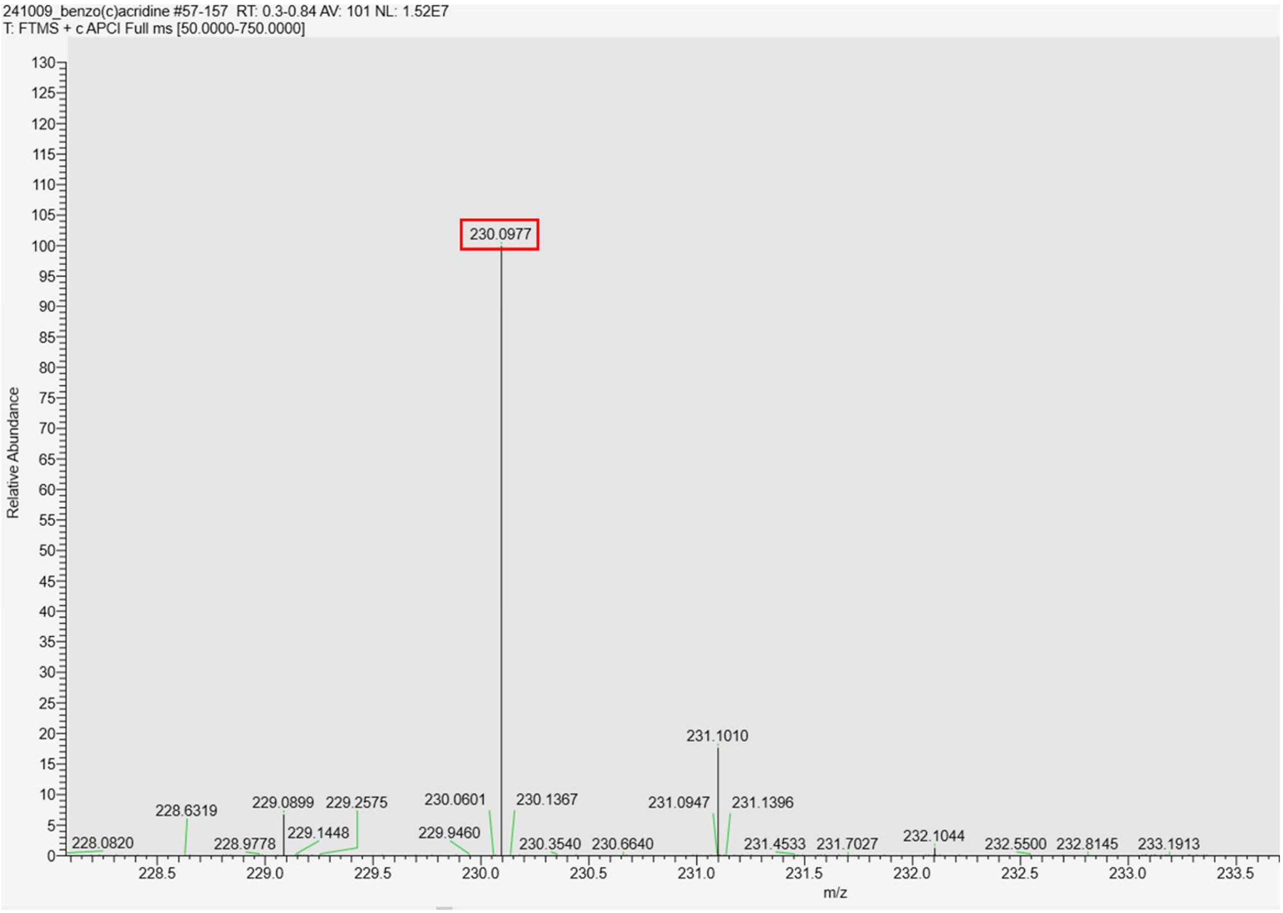


Figure S11 Mass Spectrum of Benzo(c)acridine acquired in APCI Positive mode, showing the molecular ion $[M+H]^+$ at m/z 230.0977.

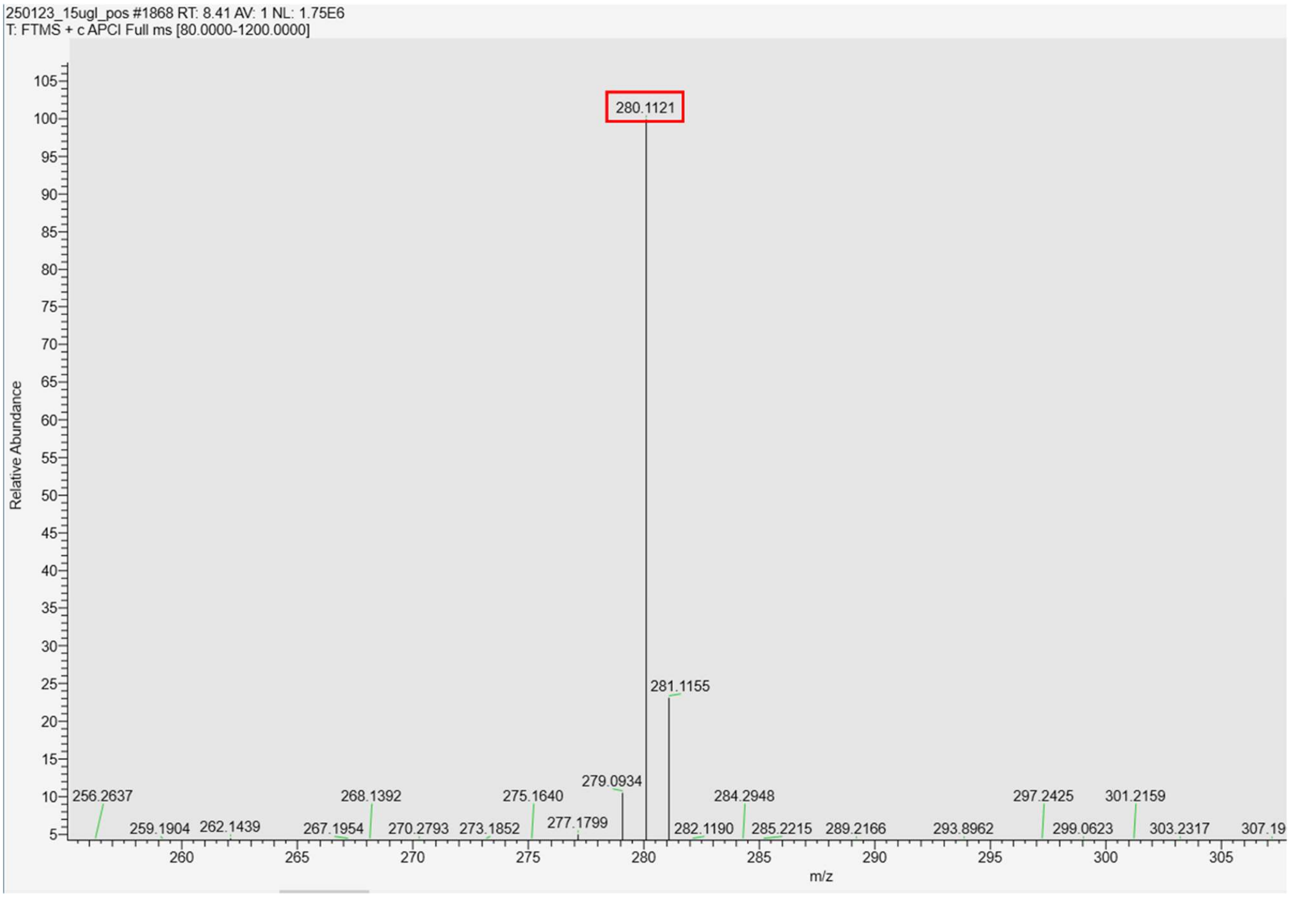


Figure S12 Mass Spectrum of Dibenzo(a,j)acridine acquired in APCI Positive mode, showing the molecular ion $[M+H]^+$ at m/z 280.1121

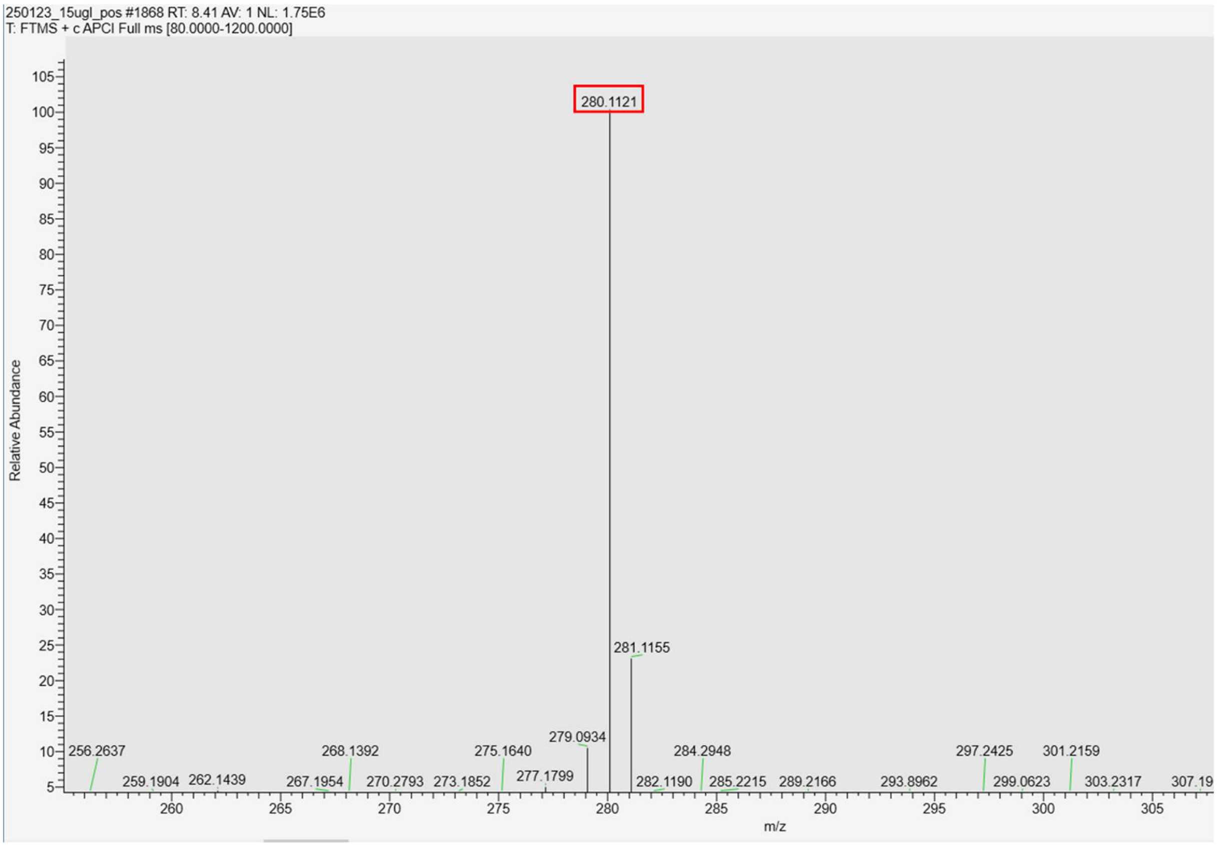


Figure S13 Mass Spectrum of Dibenzo(a,h)acridine acquired in APCI Positive mode, showing the molecular ion $[M+H]^+$ at m/z 280.1121

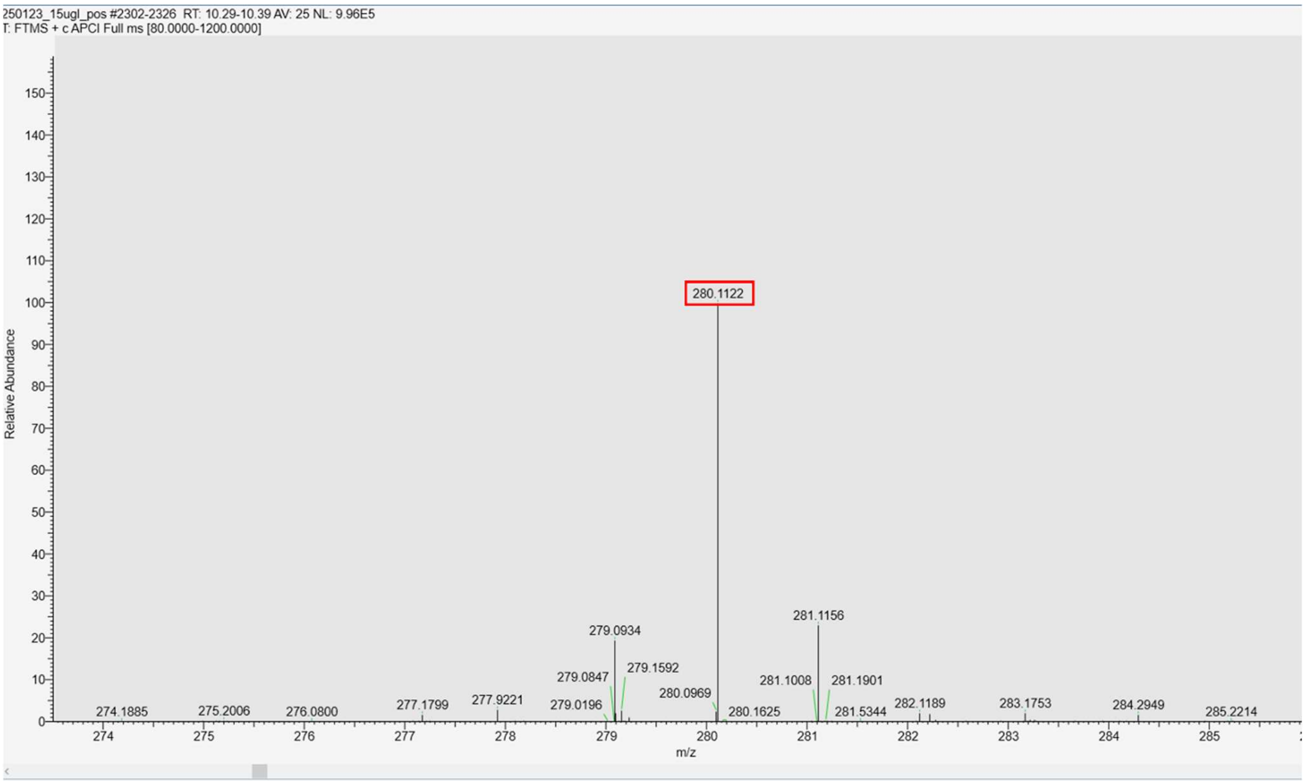


Figure S14 Mass Spectrum of Dibenzo(c,h)acridine acquired in APCI Positive mode, showing the molecular ion $[M+H]^+$ at m/z 280.1122
